# Author Correction: Disentangling oncogenic amplicons in esophageal adenocarcinoma

**DOI:** 10.1038/s41467-024-48912-y

**Published:** 2024-05-28

**Authors:** Alvin Wei Tian Ng, Dylan Peter McClurg, Ben Wesley, Shahriar A. Zamani, Emily Black, Ahmad Miremadi, Olivier Giger, Rogier ten Hoopen, Ginny Devonshire, Aisling M. Redmond, Nicola Grehan, Sriganesh Jammula, Adrienn Blasko, Xiaodun Li, Samuel Aparicio, Simon Tavaré, Oesophageal Cancer Clinical and Molecular Stratification (OCCAMS) Consortium, Paul A. W. Edwards, Nicola Grehan, Barbara Nutzinger, Christine Loreno, Sujath Abbas, Adam Freeman, Elizabeth C. Smyth, Maria O’Donovan, Ahmad Miremadi, Shalini Malhotra, Monika Tripathi, Calvin Cheah, Hannah Coles, Curtis Millington, Matthew Eldridge, Maria Secrier, Sriganesh Jammula, Jim Davies, Charles Crichton, Nick Carroll, Richard H. Hardwick, Peter Safranek, Andrew Hindmarsh, Vijayendran Sujendran, Stephen J. Hayes, Yeng Ang, Andrew Sharrocks, Shaun R. Preston, Izhar Bagwan, Vicki Save, Richard J. E. Skipworth, Ted R. Hupp, J. Robert O’Neill, Olga Tucker, Andrew Beggs, Philippe Taniere, Sonia Puig, Gianmarco Contino, Timothy J. Underwood, Robert C. Walker, Ben L. Grace, Jesper Lagergren, James Gossage, Andrew Davies, Fuju Chang, Ula Mahadeva, Vicky Goh, Francesca D. Ciccarelli, Grant Sanders, Richard Berrisford, David Chan, Ed Cheong, Bhaskar Kumar, L. Sreedharan, Simon L. Parsons, Irshad Soomro, Philip Kaye, John Saunders, Laurence Lovat, Rehan Haidry, Michael Scott, Sharmila Sothi, Suzy Lishman, George B. Hanna, Christopher J. Peters, Krishna Moorthy, Anna Grabowska, Richard Turkington, Damian McManus, Helen Coleman, Russell D. Petty, Freddie Bartlet, Karol Nowicki-Osuch, Rebecca C. Fitzgerald

**Affiliations:** 1 Early Cancer Institute, University of Cambridge, Cambridge, CB2 0XZ UK; 2 Cancer Research UK Cambridge Institute, University of Cambridge, Cambridge, UK; 3 Lee Kong Chian School of Medicine, Nanyang Technological University, Singapore, Singapore; 4 Irving Institute for Cancer Dynamics, Columbia University, New York, USA; 5 Department of Histopathology, Cambridge University Hospitals NHS Foundation Trust, Cambridge, UK; 6 Department of Pathology, University of Cambridge, Cambridge, CB2 0QQ UK; 7 Department of Oncology, University of Cambridge, Cambridge, CB2 0QQ UK; 8 Department of Molecular Oncology, British Columbia Cancer Research Centre, Vancouver, British Columbia Canada; 9 Department of Pathology and Laboratory Medicine, University of British Columbia, Vancouver, British Columbia Canada; 10 Department of Statistics, Columbia University, New York, USA; 11 Department of Biological Sciences, Columbia University, New York, USA; 12 Department of Computer Science, University of Oxford, Oxford, OX1 3QD UK; 13 Cambridge University Hospitals NHS Foundation Trust, Cambridge, CB2 0QQ UK; 14 Salford Royal NHS Foundation Trust, Salford, M6 8HD UK; 15 Faculty of Medical and Human Sciences, University of Manchester, Manchester, M13 9PL UK; 16 Wigan and Leigh NHS Foundation Trust, Wigan, Manchester WN1 2NN UK; 17 Guy’s and St Thomas’s NHS Foundation Trust, London, SE1 7EH UK; 18 GI science centre, University of Manchester, Manchester, M13 9PL UK; 19 Royal Surrey County Hospital NHS Foundation Trust, Guildford, GU2 7XX UK; 20 Edinburgh Royal Infirmary, Edinburgh, EH16 4SA UK; 21 Edinburgh University, Edinburgh, EH8 9YL UK; 22 University Hospitals Birmingham NHS Foundation Trust, Birmingham, B15 2GW UK; 23 Heart of England NHS Foundation Trust, Birmingham, B9 5SS UK; 24 Institute of Cancer and Genomic sciences, University of Birmingham, Birmingham, B15 2TT UK; 25 University Hospital Southampton NHS Foundation Trust, Southampton, SO16 6YD UK; 26 Cancer Sciences Division, University of Southampton, Southampton, SO17 1BJ UK; 27 Karolinska Institute, Stockholm, SE-171 77 Sweden; 28 King’s College London, London, WC2R 2LS UK; 29 Plymouth Hospitals NHS Trust, Plymouth, PL6 8DH UK; 30 Norfolk and Norwich University Hospital NHS Foundation Trust, Norwich, NR4 7UY UK; 31 Nottingham University Hospitals NHS Trust, Nottingham, NG7 2UH UK; 32 University College London, London, WC1E 6BT UK; 33 Wythenshawe Hospital, Manchester, M23 9LT UK; 34 University Hospitals Coventry and Warwickshire NHS, Trust, Coventry, CV2 2DX UK; 35 Peterborough Hospitals NHS Trust, Peterborough City Hospital, Peterborough, PE3 9GZ UK; 36 Department of Surgery and Cancer, Imperial College, London, W2 1NY UK; 37 Queen’s Medical Centre, University of Nottingham, Nottingham, UK; 38 Centre for Cancer Research and Cell Biology, Queen’s University Belfast, Belfast, BT7 1NN Northern Ireland; 39 Tayside Cancer Centre, Ninewells Hospital and Medical School, Dundee, DD1 9SY UK; 40 Portsmouth Hospitals NHS Trust, Portsmouth, PO6 3LY UK

**Keywords:** Cancer genomics, Oncogenes, Oesophageal cancer, Tumour heterogeneity, Cancer models

Correction to: *Nature Communications* 10.1038/s41467-024-47619-4, published online 14 May 2024

In this article, the affiliation details for Karol Nowicki-Osuch were incorrectly given as ‘Lee Kong Chian School of Medicine, Nanyang Technological University, Singapore, Singapore’ but should have been ‘Irving institute for Cancer Dynamics, Columbia University, New York, USA’. The original article has been corrected.

