# Supplementary material for: Disentangling oncogenic amplicons in esophageal adenocarcinoma
Source: Nat Commun. 2024 May 14;15:4074. doi: 10.1038/s41467-024-47619-4 (PMC11094127; doi:10.1038/s41467-024-47619-4)
Supplement: Supplementary file 1 — Supplementary Information [file 41467_2024_47619_MOESM1_ESM.pdf]

Supplementary Data

Supplementary Table 1. Cohort demographics
Supplementary Table 2. Summary of amplicon events in 710 cohort
Supplementary Table 3. Lasso regression of amplicons
Supplementary Table 4. Concordance of ecDNA classification by AA and assembly-based method in tumors
Supplementary Table 5 Organoid passage data
Supplementary Table 6. Concordance of amplicon classification by AA in tumour and organoids
Supplementary Table 7. Concordance of ecDNA classification by AA and assembly-based method in organoids

Supplementary Figure 1. Amplicons with JUP amplifications and without ERBB2 amplification
Supplementary Figure 2. Examples of long read assembly graphs
Supplementary Figure 3. P18 *CCNE1* assembly and epigenetic clustering of ecDNA reads
Supplementary Figure 4. P139 assembly and LINE1 sources and insertions
Supplementary Figure 5. Amplicon Architect reconstruction of CAM277 ecDNA circles
Supplementary Figure 6. Long read assembly graphs of organoids and methylation profiles
Supplementary Figure 7. CNV and SV events differing between passages after clonal shift in CAM277
Supplementary Figure 8. Survival analysis and methylation profiles of ecDNA containing tumours

Supplementary Table 1 Cohort Demographics

	**Overall (N=710)**
Summary statistics for Age and Stage of tumours in the OCCAMS 710 cohort	
**DI.ageAtDiagnosis**	
Mean (SD)	66.8 (9.67)
Median [Min, Max]	67.6 [25.2, 88.2]
Missing	2 (0.3%)
**pTStage_four**	
T1	98 (13.8%)
T2	70 (9.9%)
T3	311 (43.8%)
T4	44 (6.2%)
Missing	187 (26.3%)
**RP.TStage.PrimaryTumour**	
Tx	2 (0.3%)
T0	28 (3.9%)
Tis	1 (0.1%)
T1	12 (1.7%)
T1a	21 (3.0%)
T1b	64 (9.0%)
T2	70 (9.9%)
T3	311 (43.8%)
T4	9 (1.3%)
T4a	34 (4.8%)
T4b	1 (0.1%)
Missing	157 (22.1%)

Supplementary Table 2 Summary of Amplicon events in 710 tumours

Summary of driver genes amplified as an ecDNA, breakage-fusion-bridge (BFB), Complex non-cyclic amplicons and linear amplifications. Two proportions z test (prop.test in R) was used with a one sided test with alternative = 'greater'. Multiple testing was carried out using FDR correction (FDR < 0.1)

	**ecDNA**	**BFB**	**Complex non cyclic**	**Linear Amplifications**
**Number of events**	241	175	122	74
**Proportion of events**	0.3937908	0.2859477	0.1993464	0.120915

Drivers	ecDNA	BFB	Complex non-cyclic	Linear amplification	p.val*	FDR	**ecDNA (%)**	**BFB(%)**		
KRAS	25	53	4	1	0.99888273	0.99888273	0.3	0.63	83	0.117
**ERBB2**	43	25	10	4	0.019625165	**0.0588755**	0.52	0.3	82	0.115
GATA4	24	15	29	6	0.100092402	0.18507852	0.32	0.2	74	0.104
GATA6	20	13	13	19	0.148134936	0.21786638	0.31	0.2	65	0.092
CCND1	8	11	18	13	0.676822402	0.73835171	0.16	0.22	50	0.07
CDK6	20	12	8	10	0.107962469	0.18507852	0.4	0.24	50	0.07
CCNE1	14	16	8	6	0.57243393	0.68692072	0.32	0.36	44	0.062
**MYC**	24	7	7	2	0.002028568	**0.01217141**	0.6	0.17	40	0.056
CCND3	13	6	9	6	0.084334309	0.18507852	0.38	0.18	34	0.048
EGFR	16	10	3	5	0.163399784	0.21786638	0.47	0.29	34	0.048
**MDM2**	18	6	8	2	0.012372336	**0.04948934**	0.53	0.18	34	0.048
**HMGA2**	16	1	5	0	3.43E-04	**0.00411022**	0.73	0.05	22	0.031

Supplementary Table 3 Lasso regression of amplicons

Lasso regression model to identify epigenetic marks (H3K36me3, H3K4me3,H3Ka7Ac), transcription factor binding (GATA6, HNF4A, KLF), replication time and DNAse sensitivity associated with presence of amplicons.

		BFB			ecDNA		
		**Coefficient**	**Z score**	**Pr(>|Z|)**	**Coefficient**	**Z score**	**Pr(>|Z|)**
Source	(Intercept)	8.90110972	44.0831135	0	8.61120422	80.7857086	0
Encode	H3K36me3	0	0	1	0	0	1
Encode	H3K4me3	0	0	1	0	0	1
Rogerson 2019	gata6	0	0	1	0	0	1
Rogerson 2019	hnf4	0	0	1	0.15332098	2.88890832	0.00386582
Chen 2019	H3K27Ac	0	0	1	0.07656933	2.46131179	0.013843
Encode	replication-time	0.59227194	5.20030503	1.99E-07	0.18087639	2.75900325	0.0057978
Encode	dnase	7.50E-04	0.26252676	0.79291536	0	0	1
Rogerson 2020	klf	0	0	1	0	0	1

Supplementary Table 4 Concordance of ecDNA classification by AA and assemblies

Comparison of Amplicon Architect classification of amplicon type, driver gene amplified and ecAssemble assembly classification (Cyclic: Yes / No)

ID	event	Copy Number	Drivers	Type	Cyclic
P100	1	25.55418726	MET	ecDNA	Y
P100	2	6.957588637		Complex non-cyclic	N
P100	3	9.979532592	SUZ12,RARA,CCR7	ecDNA	Y
P100	4	9.94429074	ERBB2,RARA,CCR7	BFB	N
P018	5	6.75181415		Complex non-cyclic	N
P018	6	39.70815556	ERBB2,CCNE1,CIC	ecDNA	Y
P65	7	7.35867783	FOXP1	BFB	N
P65	8	41.14820135	ERBB2	ecDNA	Y
P65	9	5.661082959		Complex non-cyclic	N
P65	10	5.415782697	MYC	Complex non-cyclic	N
P139	11	16.33441927	TFEB,CCND3,GRM3,GATAD1,CDK6,KRAS	ecDNA	Y
P139	12	6.95891506		BFB	N
P139	13	5.527874239		BFB	N
P139	14	6.854239037		Complex non-cyclic	N
P113	15	10.278109		BFB	N
P43	16	4.735417258		ecDNA	N
P43	17	18.94325423	ERBB2,RARA,CCR7	ecDNA	Y
P139	18	13.94298041	GRM3,GATAD1,CDK6,KRAS	ecDNA	Y
P139	19	14.04203186	GATAD1,CDK6	ecDNA	Y

Supplementary Table 5 Organoid passage data

Organoid lines used in this study and passage numbers for each organoid

public_name	cam_id	organoid_passage
PD26661	CAM277	U
PD26661	CAM277	8
PD26661	CAM277	14
PD29271	CAM338	4
PD29271	CAM338	12
PD29269	CAM292	8
PD26660	CAM296	4
PD26660	CAM296	8
PD26660	CAM296	11
PD31009	CAM401	10
PD31009	CAM401	16
PD29268	CAM247	U
WTSI-OESO_003	CAM388	U
WTSI-OESO_009	CAM408	U
WTSI-OESO_036	CAM255	NA
WTSI-OESO_040	CAM412	U
WTSI-OESO_091	CAM429	U
WTSI-OESO_135	CAM446	NA
WTSI-OESO_088	CAM450	U
WTSI-OESO_131	CAM453	U
WTSI-OESO_095	CAM453	U
WTSI-OESO_165	CAM501	U
WTSI-OESO_197	CAM535	NA
WTSI-OESO_224	CAM576	NA

Supplementary Table 6 Concordance of amplicon classication by AA in Tumour and organoid

Comparison of Amplicon Architect classification of amplicon types and driver genes in primary tumour and organoid lines. NA : Event not identified in primary tumour, Comment: Conclusion after manual curation

Organoid ID	Driver	Organoid	Tumour	Comment
CAM247	MDM2	ecDNA	NA	No tissue
CAM247	HMGA2	ecDNA	NA	No tissue
CAM247	GATA6	ecDNA	NA	No tissue
CAM292	CDK6	others	NA	Complex non-cyclic
CAM296	MDM2	BFB	others	Complex non-cyclic
CAM296	HMGA2	BFB	others	Complex non-cyclic
CAM338	MYC	others	NA	Complex non-cyclic
CAM388	GATA6	BFB	NA	Detected only in organoid
CAM388	GATA4	BFB	BFB	Complex non-cyclic
CAM408	GATA4	BFB	NA	BFB
CAM412	CDK6	NA	BFB	No event
CAM429	CDK6	BFB	NA	BFB
CAM446	GATA6	others	NA	Others
CAM450	CDK6	others	NA	Complex non-cyclic
CAM450	CCND1	ecDNA	NA	Linear amplification
CAM453	MYC	ecDNA	NA	Detected only in organoid
CAM501	ERBB2	ecDNA	NA	No tissue
CAM535	MDM2	ecDNA	NA	ecDNA
CAM535	HMGA2	ecDNA	NA	ecDNA
CAM535	CDK6	BFB	NA	BFB
CAM535	CCND1	ecDNA	NA	ecDNA
CAM576	ERBB2	ecDNA	NA	No tissue
CAM255	MDM2	ecDNA	NA	ecDNA
CAM255	GATA6	others	NA	Linear amplification

Supplementary Table 7 Concordance of ecDNA classification by AA and assembly based methods

Comparison of Amplicon Architect classification of ecDNA and BFB events and ecAssemble assembly classification (Cyclic True / False). *events had CN < 10 and <2 reads were present at junctions of each ecDNA.

		Assembly classification	Amplicon Architect output						
	cam_id	Cyclic	AverageAmplifiedCopyCount	Oncogenes	OAC Drivers	amplicon_decomposition_class	ecDNA+	BFB+	Intervals
CAM277_ecDNA_21068	CAM277	F*	7.2047984	IFNG		Cyclic	Positive	None detected	chr12:67357001-68447185
CAM277_ecDNA_23081	CAM277	T	8.405327546			Cyclic	Positive	None detected	chr4:161160435-161181206,chr4:162827327-164198098,chr4:164657539-164668311,chr4:166845071-176425842,chr4:176567632-177538264,chr4:178702153-178712924,chr4:189727879-189938650,chr19:53175747-53206519
CAM277_ecDNA_9101	CAM277	T	46.53287757	KRAS	KRAS,LRRK2	Cyclic	Positive	None detected	chr12:24916381-25967152,chr12:40700262-41251132
CAM277_ecDNA_22317	CAM277	F	13.17165237	SDC4	HNF4A	Cyclic	None detected	Positive	chr20:41622755-43954621
CAM408_ecDNA_26604	CAM408	F	12.56101829	GATA4	GATA4	Cyclic	None detected	Positive	chr1:237142115-237152849,chr8:10210865-12111599,chr8:32897406-33579108
CAM408_ecDNA_27938	CAM408	F	13.77674171	CDK12,ERBB2,SMARCE1,RARA	ERBB2,RARA,CCR7	Cyclic	None detected	Positive	chr17:37385188-40178027
CAM408_ecDNA_25547	CAM408	F*	7.891501068	OLIG2	OLIG2	Cyclic	Positive	None detected	chr21:34030723-34592090
	CAM535	F	6.37050361			Complex non-cyclic	None detected	None detected	chr12:38455486-38486381,chr12:40322053-40342948,chr12:41282046-41986181
OG_CAM_535_ecDNA_5597	CAM535	T (L1)	15.01408553	NUMA1,CCND1,HMGA2,WIF1,MDM2,PICALM	CCND1,HMGA2,MDM2,CCND1	Cyclic	Positive	None detected	chr11:3443351-3464246,chr11:65715263-88846158,chr11:89188912-89219807,chr12:37841311-37862205,chr12:54694096-54794990,chr12:61693759-72024654,chr12:72987202-76568097,chr12:82138823-82149718
OG_CAM_535_ecDNA_7973	CAM535	F	23.1828722	AKAP9,CDK6	GATAD1,CDK6	Cyclic	None detected	Positive	chr7:90696720-92783329
	CAM535	F	6.497473149	HOXC11,HOXC13	HOXC13,HOXC11	Complex non-cyclic	None detected	None detected	chr11:71498877-71509771,chr11:89207845-89218739,chr12:54285673-55133432
OG_CAM_535_ecDNA_20799	CAM535	F*	10.4142474	PPARG,XPC,SRGAP3,VHL,FANCD2,RAF1	RAF1	Cyclic	Positive	None detected	chr3:8643250-14494145,chr3:15097718-15937110,chr10:75319559-75330453

A

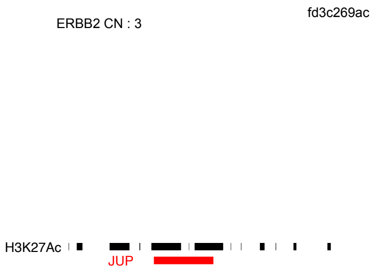
ERBB2 CN : 3
fd3c269ac
H3K27Ac
JUP

B

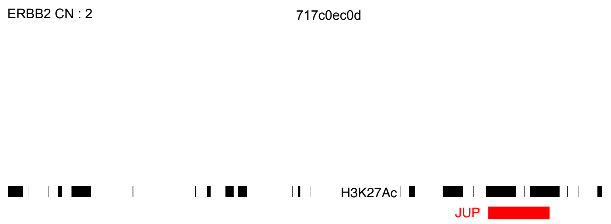
ERBB2 CN : 2
717c0ec0d
H3K27Ac
JUP

C

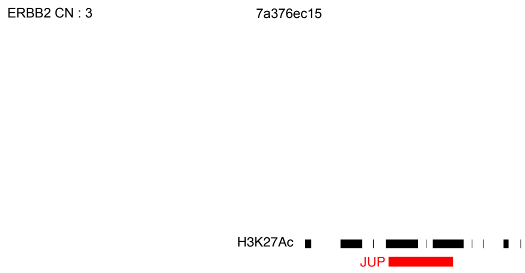
ERBB2 CN : 3
7a376ec15
H3K27Ac
JUP

D

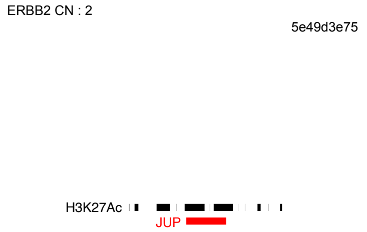
ERBB2 CN : 2
5e49d3e75
H3K27Ac
JUP

E

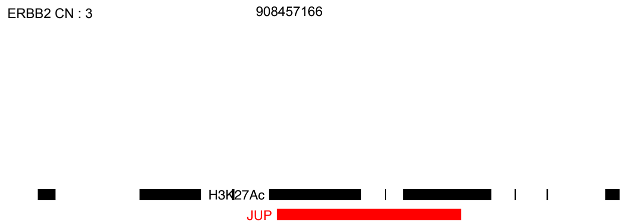
ERBB2 CN : 3
908457166
H3K27Ac
JUP

F

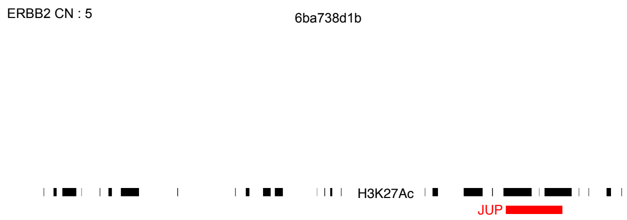
ERBB2 CN : 5
6ba738d1b
H3K27Ac
JUP

G

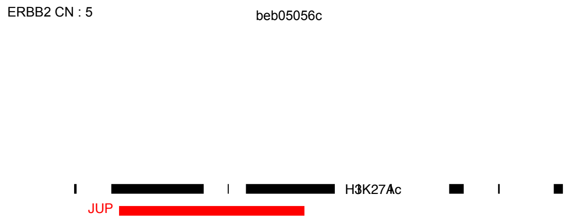
ERBB2 CN : 5
beb05056c
H3K27Ac
JUP

Supplementary Figure 1. A-G) Amplicons with JUP amplifications and without ERBB2 amplification. Copy number plots showing amplified regions in each tumor and lines denote split reads between regions. H3K27Ac track denotes putative enhancer regions (black) and *JUP* gene region is highlighted in red. Copy number of ERRB2 determined using CNVKit.

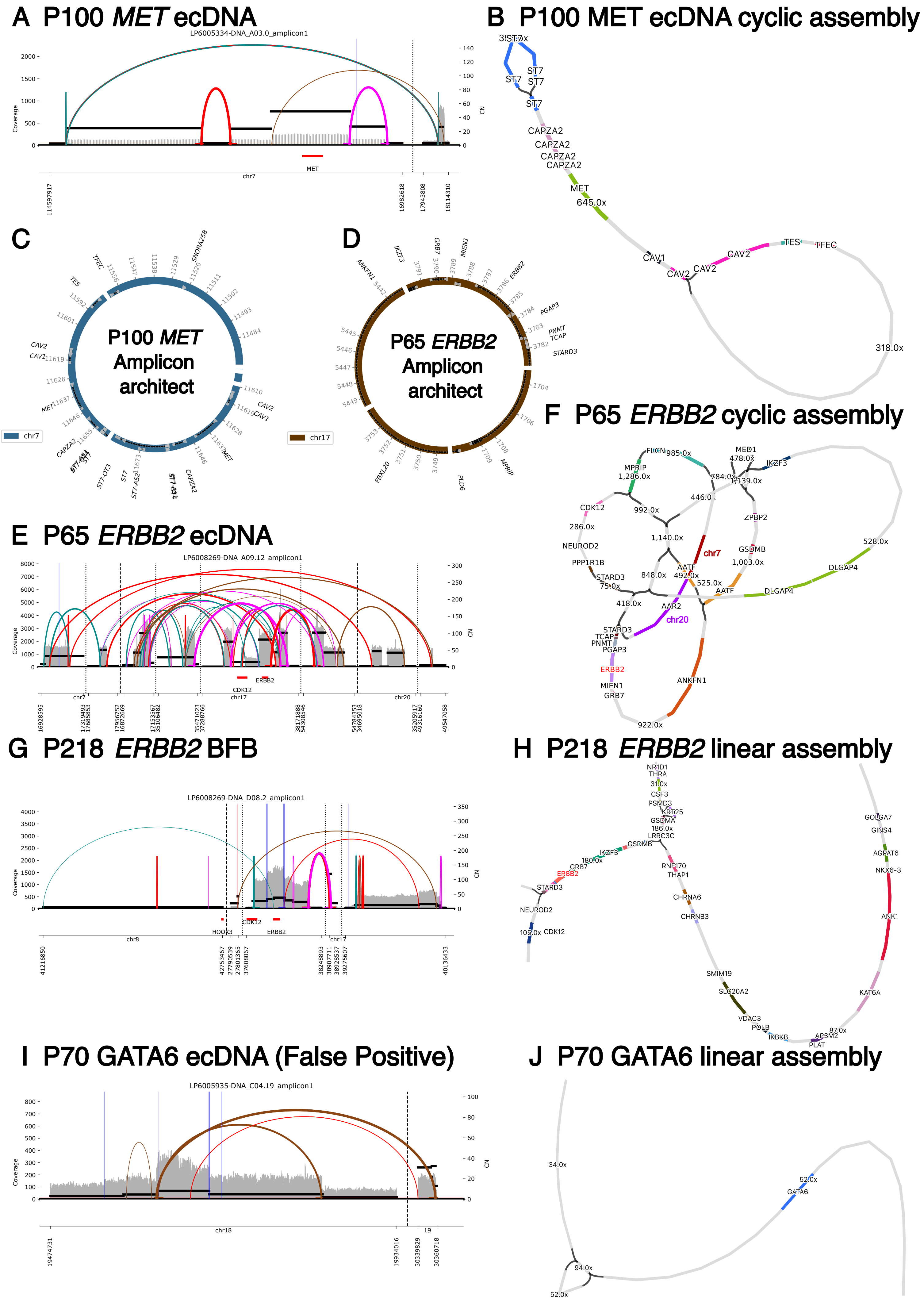
A P100 MET ecDNA
LP6005334-DNA_A03.0_amplicon1
Coverage
CN
MET
chr7
B P100 MET ecDNA cyclic assembly
ST7
CAPZA2
MET
645.0x
CAV1
CAV2
TES
TFEC
318.0x
C
P100 MET Amplicon architect
chr7
D
P65 ERBB2 Amplicon architect
chr17
E P65 ERBB2 ecDNA
LP6008269-DNA_A09.12_amplicon1
Coverage
CN
ERBB2
CDK12
chr7
chr17
chr20
F P65 ERBB2 cyclic assembly
FLCN
985.0x
MED1
478.0x
IKZF3
MPRIP
1,286.0x
784.0x
1,139.0x
CDK12
992.0x
446.0x
ZPBP2
286.0x
1,140.0x
528.0x
NEUROD2
chr7
GSDMB
PPP1R1B
AATF
1,003.0x
DLGAP4
STARD3
848.0x
492.0x
75.0x
525.0x
AATF
DLGAP4
418.0x
AAR2
chr20
STARD3
TCAP
PNMT
PGAP3
ERBB2
ANKFN1
MIEN1
GRB7
922.0x
G P218 ERBB2 BFB
LP6008269-DNA_D08.2_amplicon1
Coverage
CN
CDK12
HOOK3
ERBB2
chr8
chr17
H P218 ERBB2 linear assembly
NR1D1
THRA
31.0x
CSF3
PSMD3
KRT25
GSDMA
186.0x
LRRC3C
GSDMB
IKZF3
180.0x
GRB7
ERBB2
STARD3
NEUROD2
105.0x
CDK12
RNF170
THAP1
CHRNA6
CHRNB3
SMIM19
SLC20A2
VDAC3
POLB
IKBKB
AP3M2
PLAT
87.0x
KAT6A
ANK1
NKX6-3
AGPAT6
GINS4
GOLGA7
I P70 GATA6 ecDNA (False Positive)
LP6005935-DNA_C04.19_amplicon1
Coverage
CN
chr18
19
J P70 GATA6 linear assembly
34.0x
52.0x
GATA6
94.0x
52.0x

Supplementary Figure 2. Examples of long read assembly graphs. A) Copy number plot of *MET* ecDNA in P100. B) Cyclic assembly of the *MET* with genes annotated. C) Amplicon Architect (AA) reconstruction of the P100 *MET* ecDNA showing concordant structures. D) AA reconstruction of *ERBB2* ecDNA in P65. E) Copy number plot of *ERBB2* ecDNA in P65. F) Cyclic assembly of P65 containing ERBB2 ecDNA highlighted in red. G) Copy number plot of *ERBB2* BFB in P218 and H) linear assembly graph of the region. I) Copy number plot of a false positive event in P70 near *GATA6* and J) linear assembly graph. Of note, *GATA6* was outside the cyclic region and the genomic region on chr19 show evidence of a putative enhancer element.

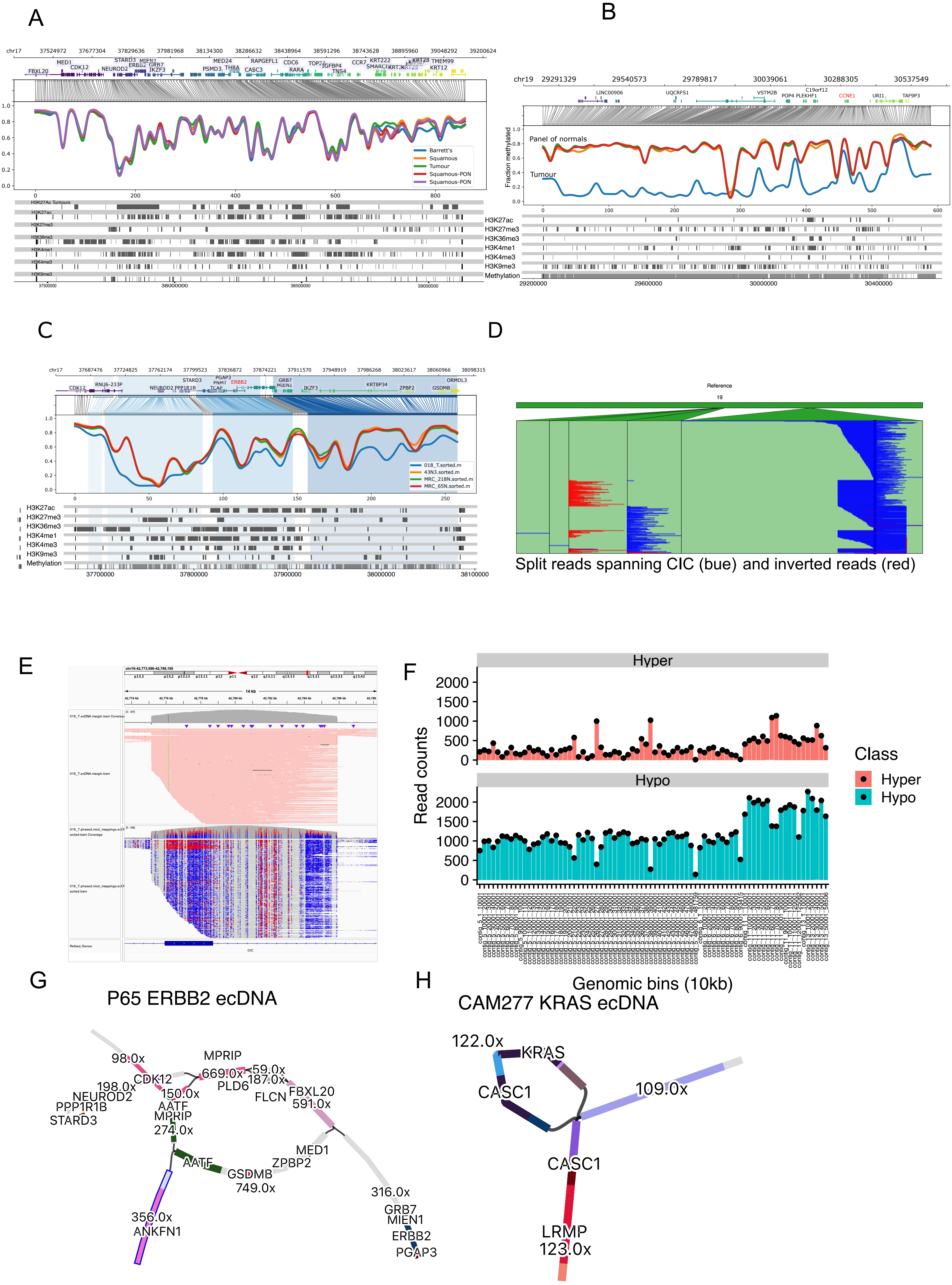
A
B
C
D
Split reads spanning CIC (bue) and inverted reads (red)
E
F
Hyper
Hypo
Read counts
Class
Hyper
Hypo
Genomic bins (10kb)
G
P65 ERBB2 ecDNA
H
CAM277 KRAS ecDNA
KRAS
CASC1
LRMP
122.0x
109.0x
123.0x

Supplementary Figure 3. P18 *CCNE1* assembly and epigenetic clustering of ecDNA reads. A) Methylation of 43B and 43T compared to a paired normal squamous and two panel of normal tissue. Annotation from Chen et al and the Epigenome Roadmap shown as tracks below. B) Methylation profile of the amplified regions near *CCNE1* showing fraction of reads methylated with gene annotations included above. Epigenome roadmap annotations of the region and differential methylated regions (DMR) based on a panel of three normal squamous tissues shown below. C) Methylation profile of amplified regions in *ERBB2* BFB in P100. Highlighted regions show DMR compared to a panel of normals. D) Ribbon plot of split reads mapping to *CIC* containing region. Inverted reads are shown in red. E) IGV screenshot of *CIC* region showing the haplotype phased bam (top) and methylated reads (bottom). Methylated reads (red) and hypomethylated reads (blue) mapping to the *CIC* exon show differential methylation between unclipped reads mapping to the BFB and clipped reads mapping to the ecDNA. F) Read counts of hypomethylated and hypermethylated reads in each 10Kb genomic bin in the assembly graph. G) Refined assembly of ERBB2 containing amplicon 65T using hypomethylated reads. H) Refined assembly of KRAS containing amplicon in CAM277.

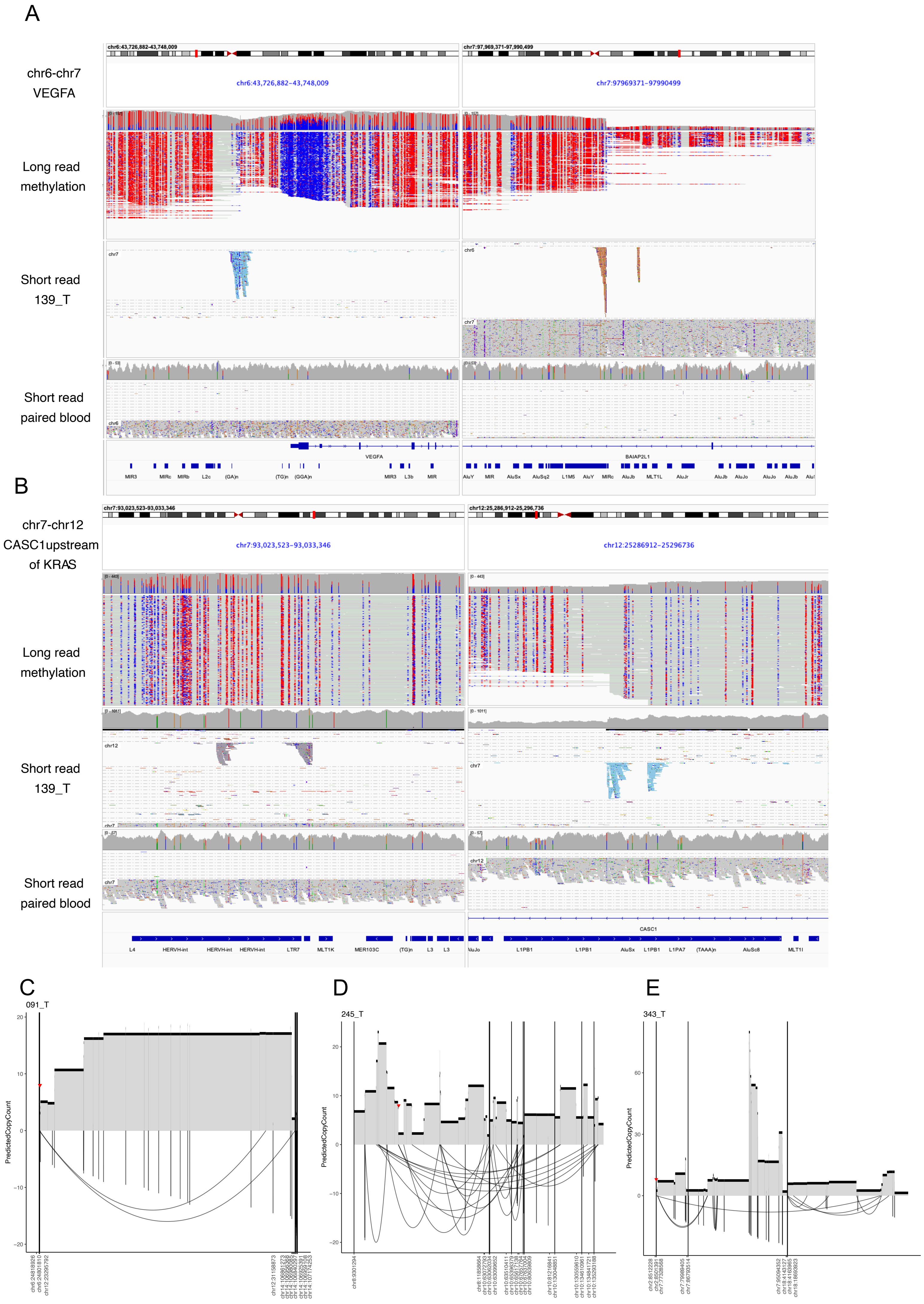

A
chr6-chr7
VEGFA
chr6:43,726,882-43,748,009
chr7:97,969,371-97,990,499
chr6:43,726,882-43,748,009
chr7:97969371-97990499
Long read
methylation
Short read
139_T
chr7
chr6
Short read
paired blood
VEGFA
BAIAP2L1
B
chr7-chr12
CASC1upstream
of KRAS
chr7:93,023,523-93,033,346
chr12:25,286,912-25,296,736
chr7:93,023,523-93,033,346
chr12:25286912-25296736
Long read
methylation
Short read
139_T
chr12
chr7
Short read
paired blood
CASC1
C
091_T
PredictedCopyCount
D
245_T
PredictedCopyCount
E
343_T
PredictedCopyCount

Supplementary Figure 4. **P139 assembly and LINE1 sources and insertions.** A) IGV screenshot of the LINE-1 insertion at the *VEGFA* locus with long reads sequencing with methylation annotation (top), short read WGS of the tumor (middle) and short read WGS of normal blood from the same patient (bottom). Mapping region of the paired mate of the reads show a drop in coverage and supplementary reads linking between chr7 (light blue) and chr6 (orange). B) IGV screenshot of LINE-1 insertion at the *CASC1* locus upstream of *KRAS.* Supplementary reads linking chr12 and chr7 shown in grey and light blue respectively. C-E) Example of a BFB event and amplicon events with L1 insertion < 50kb from the amplicon.

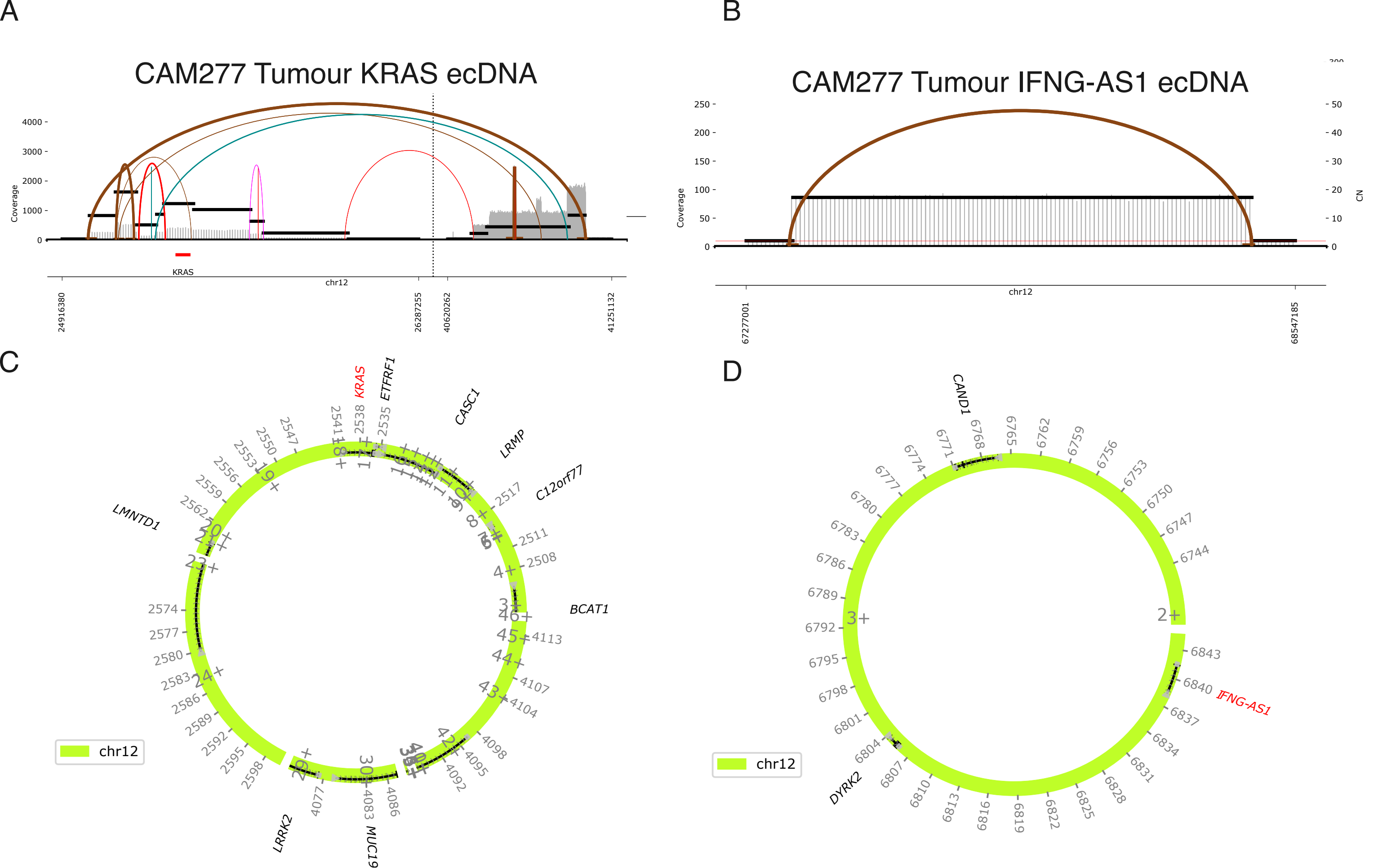

A
CAM277 Tumour KRAS ecDNA
Coverage
KRAS
chr12
24916380
26287255
40620262
41251132
B
CAM277 Tumour IFNG-AS1 ecDNA
Coverage
CN
chr12
67277001
68547185
C
KRAS
ETFRF1
CASC1
LRMP
C12orf77
BCAT1
LMNTD1
LRRK2
MUC19
chr12
D
CAND1
IFNG-AS1
DYRK2
chr12

Supplementary Figure 5. Amplicon Architect reconstruction of CAM277 ecDNA circles. Copy number plots of CAM277 tumor for A) *KRAS* and B) *IFNG-AS1* ecDNA. C-D) AA reconstruction of events based on regions amplified.

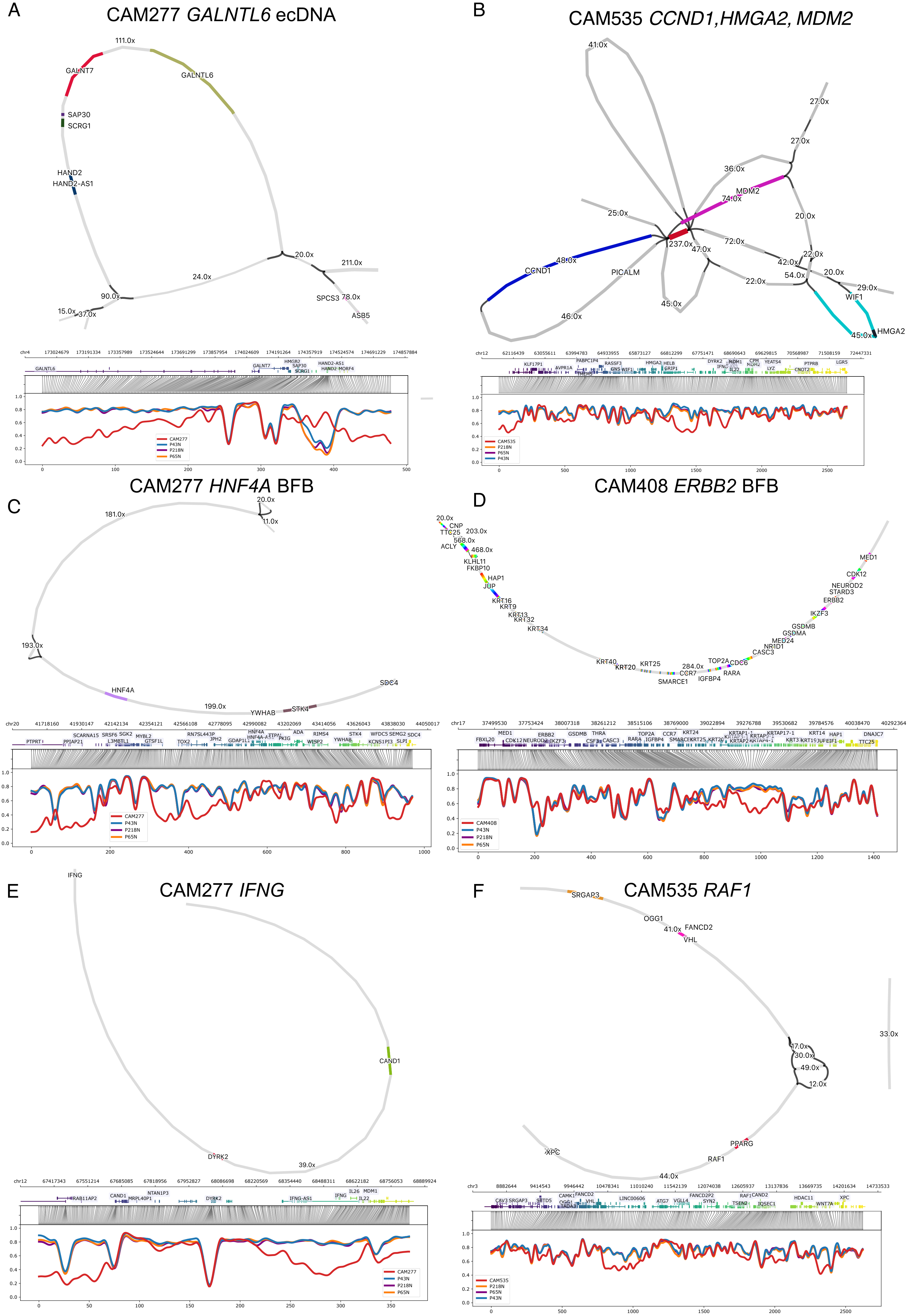
A
CAM277 GALNTL6 ecDNA
111.0x
GALNT7
GALNTL6
SAP30
SCRG1
HAND2
HAND2-AS1
20.0x
211.0x
24.0x
90.0x
15.0x
37.0x
SPCS3
78.0x
ASB5
CAM277
P43N
P218N
P65N
B
CAM535 CCND1,HMGA2, MDM2
41.0x
27.0x
27.0x
36.0x
MDM2
74.0x
25.0x
20.0x
237.0x
47.0x
72.0x
48.0x
CCND1
PICALM
42.0x
22.0x
54.0x
20.0x
22.0x
29.0x
WIF1
45.0x
46.0x
45.0x
HMGA2
CAM535
P218N
P65N
P43N
C
CAM277 HNF4A BFB
20.0x
181.0x
11.0x
193.0x
HNF4A
SDC4
199.0x
YWHAB
STK4
CAM277
P43N
P218N
P65N
D
CAM408 ERBB2 BFB
20.0x
CNP
TTC25
203.0x
568.0x
ACLY
468.0x
KLHL11
FKBP10
HAP1
JUP
KRT16
KRT9
KRT13
KRT32
KRT34
MED1
CDK12
NEUROD2
STARD3
ERBB2
IKZF3
GSDMB
GSDMA
MED24
NR1D1
CASC3
KRT40
KRT20
KRT25
284.0x
TOP2A
CDC6
CCR7
RARA
SMARCE1
IGFBP4
CAM408
P43N
P218N
P65N
E
IFNG
CAM277 IFNG
CAND1
DYRK2
39.0x
CAM277
P43N
P218N
P65N
F
SRGAP3
CAM535 RAF1
OGG1
41.0x
FANCD2
VHL
33.0x
17.0x
30.0x
49.0x
12.0x
PPARG
XPC
RAF1
44.0x
CAM535
P218N
P65N
P43N

Supplementary Figure 6. Long read assembly graphs of organoids and methylation profiles. A-B) Cyclic assembly graphs from CAM277 and CAM535 in ecDNA predicted by AA. Methylation profiles of each region shown with comparison with a panel of normal squamous tissues (P43N, P218N and P65N). C-D) BFB assembly graphs showing linear graphs. E-F) Discrepant amplicons classified by AA to be ecDNA. E) Amplicon containing *IFNG* and *IFNG-AS1* was found to decrease after organoid derivation and limit the detection of the amplicon. F) Amplicon predicted to be ecDNA by AA with <2 reads spanning distal ends of the segments.

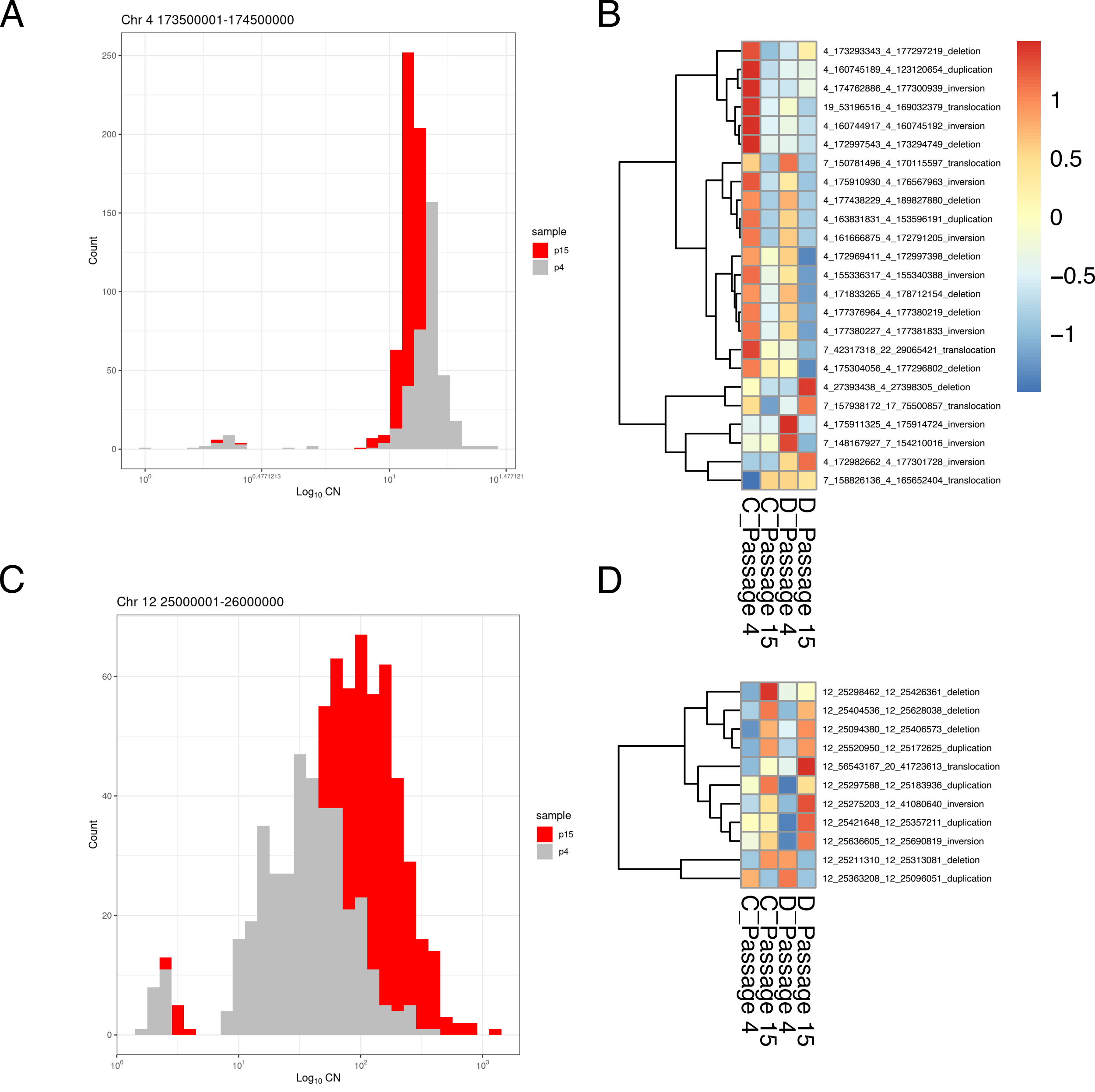

A
Chr 4 173500001-174500000
Count
Log10 CN
sample
p15
p4
B
4_173293343_4_177297219_deletion
4_160745189_4_123120654_duplication
4_174762886_4_177300939_inversion
19_53196516_4_169032379_translocation
4_160744917_4_160745192_inversion
4_172997543_4_173294749_deletion
7_150781496_4_170115597_translocation
4_175910930_4_176567963_inversion
4_177438229_4_189827880_deletion
4_163831831_4_153596191_duplication
4_161666875_4_172791205_inversion
4_172969411_4_172997398_deletion
4_155336317_4_155340388_inversion
4_171833265_4_178712154_deletion
4_177376964_4_177380219_deletion
4_177380227_4_177381833_inversion
7_42317318_22_29065421_translocation
4_175304056_4_177296802_deletion
4_27393438_4_27398305_deletion
7_157938172_17_75500857_translocation
4_175911325_4_175914724_inversion
7_148167927_7_154210016_inversion
4_172982662_4_177301728_inversion
7_158826136_4_165652404_translocation
C_Passage 4
C_Passage 15
D_Passage 4
D_Passage 15
C
Chr 12 25000001-26000000
Count
Log10 CN
sample
p15
p4
D
12_25298462_12_25426361_deletion
12_25404536_12_25628038_deletion
12_25094380_12_25406573_deletion
12_25520950_12_25172625_duplication
12_56543167_20_41723613_translocation
12_25297588_12_25183936_duplication
12_25275203_12_41080640_inversion
12_25421648_12_25357211_duplication
12_25636605_12_25690819_inversion
12_25211310_12_25313081_deletion
12_25363208_12_25096051_duplication
C_Passage 4
C_Passage 15
D_Passage 4
D_Passage 15

Supplementary Figure 7. CNV and SV events differing between passages after clonal shift in CAM277. A) Histogram showing CN values of cells at P4 and P15 in a region with a complex amplicon on chr4 and B) Heatmap of SV events of the chr4 complex amplicon, C) Histogram of a genomic bin on chr12 showing a higher CN at P15 and D) SV events on chr12 enriched at P15.

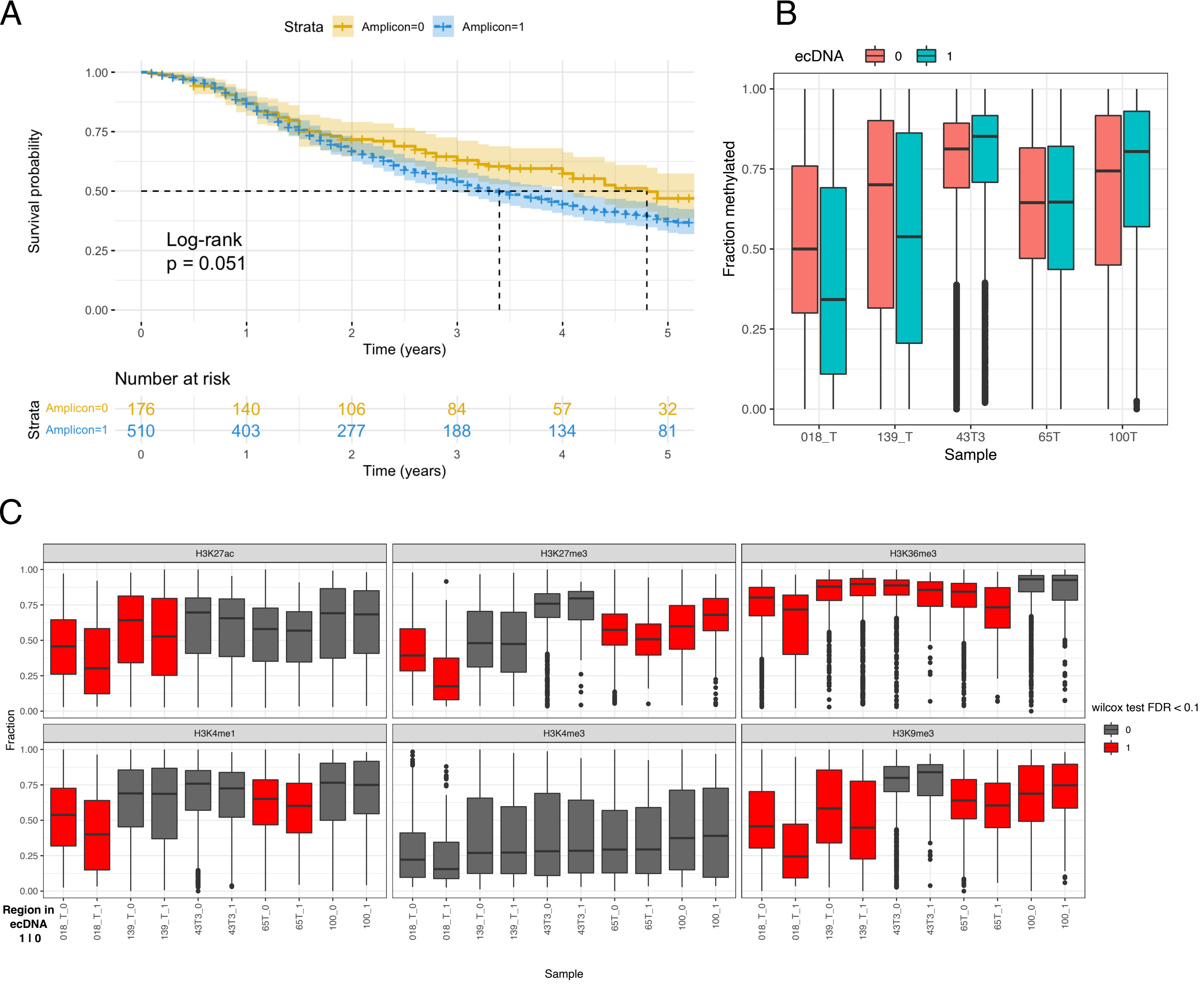
A
Strata Amplicon=0 Amplicon=1
Survival probability
Log-rank
p = 0.051
Time (years)
Number at risk
Strata
Amplicon=0 176 140 106 84 57 32
Amplicon=1 510 403 277 188 134 81
B
ecDNA 0 1
Fraction methylated
018_T 139_T 43T3 65T 100T
Sample
C
H3K27ac
H3K27me3
H3K36me3
H3K4me1
H3K4me3
H3K9me3
Fraction
wilcox test FDR < 0.1
Region in ecDNA 1 | 0
Sample

Supplementary Figure 8. Survival analysis and methylation profiles of ecDNA containing tumours. A) Kaplan Meier plots of patients in the 710 with an amplicon (BFB or ecDNA) compared with patients without amplicon events. B) Box plot showing fraction of methylation in each tumour in a union set of ecDNA regions in the five genomes and annotated if regions are part of an ecDNA amplicon in each tumour. C) Boxplot showing fraction of methylation of each tumour split by epigenome roadmap annotations (H3K27Ac,H3K4Me1,H3K27me3, H3K36me3 and H3K9Me3) Boxplots are coloured red if mean fraction methylation is different between ecDNA and non-ecDNA regions in each tumour (FDR-adjusted Wilcox test < 0.1).

The minima of the boxplot shows the lowest data point (0th percentile) and maxima shows the largest data point (100th percentile). The lower bound of the boxplot is the 25th percentile and the upper bound is 7th percentile. The centre shows the 50th percentile or median value.The upper whisker extends no long than 1.5 * Interquartile range (IQR) for the upper bound of the box and lower whisker extends no longer than 1.5 * IQR from the lower bound.
