# Supplementary material for: Disentangling oncogenic amplicons in esophageal adenocarcinoma
Source: Nat Commun. 2024 May 14;15:4074. doi: 10.1038/s41467-024-47619-4 (PMC11094127; doi:10.1038/s41467-024-47619-4)
Supplement: Supplementary file 3 — Description of Additional Supplementary Files [file 41467_2024_47619_MOESM3_ESM.pdf]

File Name: Supplementary Data 1
Description: Clinical information of the OCCAMS 710 cohort including treatment and exposure information.

File Name: Supplementary Data 2
Description: ecAssemble code used for de-novo assembly of long read sequences.
