# Supplementary material for: Disentangling oncogenic amplicons in esophageal adenocarcinoma
Source: Nat Commun. 2024 May 14;15:4074. doi: 10.1038/s41467-024-47619-4 (PMC11094127; doi:10.1038/s41467-024-47619-4)
Supplement: Supplementary file 6 — Reporting Summary [file 41467_2024_47619_MOESM6_ESM.pdf]

nature portfolio

Corresponding author(s): Rebecca Fitzgerald, Alvin Ng

Last updated by author(s): Apr 2, 2024

# Reporting Summary

Nature Portfolio wishes to improve the reproducibility of the work that we publish. This form provides structure for consistency and transparency in reporting. For further information on Nature Portfolio policies, see our Editorial Policies and the Editorial Policy Checklist.

## Statistics

For all statistical analyses, confirm that the following items are present in the figure legend, table legend, main text, or Methods section.

| n/a | Confirmed | |
|---|---|---|
| [ ] | [x] | The exact sample size (*n*) for each experimental group/condition, given as a discrete number and unit of measurement |
| [x] | [ ] | A statement on whether measurements were taken from distinct samples or whether the same sample was measured repeatedly |
| [ ] | [x] | The statistical test(s) used AND whether they are one- or two-sided<br>*Only common tests should be described solely by name; describe more complex techniques in the Methods section.* |
| [ ] | [x] | A description of all covariates tested |
| [ ] | [x] | A description of any assumptions or corrections, such as tests of normality and adjustment for multiple comparisons |
| [ ] | [x] | A full description of the statistical parameters including central tendency (e.g. means) or other basic estimates (e.g. regression coefficient) AND variation (e.g. standard deviation) or associated estimates of uncertainty (e.g. confidence intervals) |
| [ ] | [x] | For null hypothesis testing, the test statistic (e.g. *F*, *t*, *r*) with confidence intervals, effect sizes, degrees of freedom and *P* value noted<br>*Give P values as exact values whenever suitable.* |
| [x] | [ ] | For Bayesian analysis, information on the choice of priors and Markov chain Monte Carlo settings |
| [x] | [ ] | For hierarchical and complex designs, identification of the appropriate level for tests and full reporting of outcomes |
| [x] | [ ] | Estimates of effect sizes (e.g. Cohen's *d*, Pearson's *r*), indicating how they were calculated |

*Our web collection on statistics for biologists contains articles on many of the points above.*

## Software and code

Policy information about availability of computer code

| | |
|---|---|
| Data collection | Long read sequencing data was generated using Guppy 5.0.11 to base call and Megalodon 2.4.2 was used to generate the methylation calling. |
| Data analysis | Short read sequences were aligned using BWA-mem(V.07.17), CNVs called using CNVKit v0.9.8, QDNAseq 1.18.0 and Hatchet v2.0. SVs were called using Manta v0.27, Sniffles 2.06 and GRIDSS v2.7.4, LINX v1.7. ecDNA were called using Amplicon Architect v1.2, classified using Amplicon Classifier v0.4.13 and assembled using ecAssembler (https://github.com/fitzgerald-lab/ecAssemble) that was written for this study. ecAssembler uses dependencies including Flye 2.9.3-b1797, CVLR v0.1, bedtools v2.27.1 and minimap2 v2.26-r1175. Minigraph v0.2.0, TLDR v0.1 were used for long read analyses. TrimGalore v0.6.6, FastQC v0.11.9, FastQScreen v0.14.0, HBDScan v0.8.29-1, HMMCopy v0.0.23 and destruct v0.4.22 were used to process and analyze the scDNA dataset. |

For manuscripts utilizing custom algorithms or software that are central to the research but not yet described in published literature, software must be made available to editors and reviewers. We strongly encourage code deposition in a community repository (e.g. GitHub). See the Nature Portfolio guidelines for submitting code & software for further information.

# Data

Policy information about availability of data

All manuscripts must include a data availability statement. This statement should provide the following information, where applicable:

- Accession codes, unique identifiers, or web links for publicly available datasets
- A description of any restrictions on data availability
- For clinical datasets or third party data, please ensure that the statement adheres to our policy

The sequencing data included in this study have been submitted to European Genome-phenome Archive (EGA; https://ega-archive.org/) under the accession numbers EGAD00001007785 (https://ega-archive.org/datasets/EGAD00001007785) and EGAD00001006083 (https://ega-archive.org/datasets/EGAD00001006083) respectively. The raw sequencing data are available under restricted access due to data privacy laws for sensitive controlled genomic data; access can be requested to the ICGC Data Access Compliance Office as described here: https://docs.icgc-argo.org/docs/data-access/daco/applying. Applicants must be affiliated with a legal entity and submit a project summary that conforms with policies concerning the purpose of the research, protection of the donors and security of the data. Once the application has been submitted, the ICGC DACO committee will review your application and you will hear back within ten business days. Tha access to the controlled data will be granted for a period of two years. Processed data to reproduce Figure 2A is available from Zenodo (https://zenodo.org/records/10775258). Genome annotations with replication timing, DNase I accessibility and ChIP-seq from Encode21 (H3K36me3, H3K27ac, H3K4Me3, https://www.encodeproject.org/) and experimental data18,22,23 (GATA6, and HNF4A: https://www.ncbi.nlm.nih.gov/pmc/articles/PMC6499311/bin/supp_gr.243345.118_Supplemental_Table_S3.xlsx, KLF:https://cdn.elifesciences.org/articles/57189/elife-57189-supp5-v2.xlsx, H3K27Ac:https://www.ncbi.nlm.nih.gov/pmc/articles/PMC8108390/bin/NIHMS1695582-supplement-Supplementary_Tables.xlsx in tumors and cell lines) were used for lasso regression. Previously published ChIP-seq data from tumors and cell lines18 and esophagus cell line E079 from Epigenome Roadmap21 (https://egg2.wustl.edu/roadmap/web_portal/index.html) were used for additional annotations of regulatory elements including enhancer and heterochromatin elements. Previous short read sequencing data of organoid14 were used to identify clonal shifts (https://www.ebi.ac.uk/ega/datasets/EGAD00001004007). Source data are provided with this paper. 'The remaining data are available within the Article, Supplementary Information or Source Data file.

# Research involving human participants, their data, or biological material

Policy information about studies with human participants or human data. See also policy information about sex, gender (identity/presentation), and sexual orientation and race, ethnicity and racism.

| | |
|---|---|
| Reporting on sex and gender | The OCCAMS (Oesophageal Cancer Classification and Molecular Stratification) cohort consists of 603 males and 104 females and sex and gender was not considered in the study design. Self reported gender was collected as clinical information in the OCCAMS and consent has been obtained as per approval for the OCCAMS study. This information is used to describe the cohort demographics (Supplementary Table 1-2) which is predominantly male as described in previous studies.Gender based analyses have not been done as EAC has a high male dominance and an analysis on female cancers would likely be underpowered given the available data. |
| Reporting on race, ethnicity, or other socially relevant groupings | Age and ethnicity were collected as part of the clinical information and used to describe the cohort. Variables were self reported by participants and we did not do any analyses based on groups of age and ethnicity. |
| Population characteristics | This is described in Supplementary table 1 |
| Recruitment | Participants were recruited as part of the OCCAMS study from multiple centres in the UK. The participation of patients were subjected to consent to the study and the collection of biological tissue for further analyses, including genomic sequencing of tumour and healthy samples. |
| Ethics oversight | This study complies with all relevant ethical regulations. The study was approved by the Cambridge South Research Ethics Committee (REC 07/H0305/52 and 10/H0305/1) and included written individual informed consent. |

Note that full information on the approval of the study protocol must also be provided in the manuscript.

# Field-specific reporting

Please select the one below that is the best fit for your research. If you are not sure, read the appropriate sections before making your selection.

☒ Life sciences ☐ Behavioural & social sciences ☐ Ecological, evolutionary & environmental sciences

For a reference copy of the document with all sections, see nature.com/documents/nr-reporting-summary-flat.pdf

# Life sciences study design

All studies must disclose on these points even when the disclosure is negative.

| | |
|---|---|
| Sample size | All samples with available biological materials were included and no sample size calculations were carried out. This is a cross sectional study to describe the molecular features of esophageal tumours in a well powered dataset. |
| Data exclusions | A pathological review of tissue samples was done to only include samples with >= 70% tissue cellularity for the sequencing study. In addition, this study only describes tumours that have not undergone treatment or is treatment naive. No post treatment or metastatic tumours were included in this study. |

| | |
|---|---|
| Replication | We performed short read and long reads experiments independently to replicate the complex amplifications on different sequencing platforms and in different systems, e.g. patient tissue and organoid models. As the study is based on scarce human tissue samples, the experiments are limited by sample available and replicates may not be always available. The FISH micrographs were generated in replicates to ensure that the observations are replicated |
| Randomization | As this is a cross sectional study, no randomization was carried out as we reported the overall frequencies of each observation. |
| Blinding | Blinding is not relevant in our study as we aim to describe the genomic profiles of esophageal tumours in a cross sectional study. We correlated our findings to additional molecular features that can provide information to understand the underlying biological processes in these tumours. |

# Reporting for specific materials, systems and methods

We require information from authors about some types of materials, experimental systems and methods used in many studies. Here, indicate whether each material, system or method listed is relevant to your study. If you are not sure if a list item applies to your research, read the appropriate section before selecting a response.

## Materials & experimental systems

| n/a | Involved in the study |
|---|---|
| ☒ | ☐ Antibodies |
| ☒ | ☐ Eukaryotic cell lines |
| ☒ | ☐ Palaeontology and archaeology |
| ☒ | ☐ Animals and other organisms |
| ☒ | ☐ Clinical data |
| ☒ | ☐ Dual use research of concern |
| ☒ | ☐ Plants |

## Methods

| n/a | Involved in the study |
|---|---|
| ☒ | ☐ ChIP-seq |
| ☒ | ☐ Flow cytometry |
| ☒ | ☐ MRI-based neuroimaging |
